# Crystal structure of *fac*-tricarbon­yl(quinoline-2-carboxyl­ato-κ^2^ *N*,*O*)(tri­phenyl­arsane-κ*As*)rhenium(I)

**DOI:** 10.1107/S2056989015024640

**Published:** 2016-01-06

**Authors:** Charalampos Triantis, Antonio Shegani, Christos Kiritsis, Catherine P. Raptopoulou, Vassilis Psycharis, Maria Pelecanou, Ioannis Pirmettis, Minas Papadopoulos

**Affiliations:** a Institute of Nuclear and Radiological Sciences and Technology, Energy and Safety, National Centre for Scientific Research "Demokritos", 15310 Athens, Greece; b Institute of Nanoscience and Nanotechnology, National Centre for Scientific Research "Demokritos", 15310 Athens, Greece; c Institute of Biosciences & Applications, National Centre for Scientific Research "Demokritos", 15310 Athens, Greece

**Keywords:** crystal structure, rhenium(I) tricarbonyl complex, rhenium(I) tri­phenyl­arsane and quinaldic acid complex, *trans* influence

## Abstract

In the title compound, [Re(C_10_H_6_NO_2_)(CO)_3_{As(C_6_H_5_)_3_}], the coordination environment of Re^I^ is that of a distorted octa­hedron. Three coordination sites are occupied by three carbonyl groups in a facial arrangement and the remaining three sites by tri­phenyl­arsane and deprotonated quinaldic acid in *As*-mono- and *N*,*O*-bidentate fashions, respectively. In the crystal, the complexes are linked through weak C—H⋯O hydrogen bonds, forming a three-dimensional network. It worth noting that, as far as we know, this complex is the first Re^I^ tri­phenyl­arsane tricarbonyl compound to be reported.

## Abstract 2

In the title rhenium(I) tricarbonyl complex with tri­phenyl­arsane and deprotonated quinaldic acid ligands, the Re^I^ atom is in an octa­hedral coordination. Weak C—H⋯O inter­actions lead to a three-dimensional supra­molecular architecture.

## Chemical context   

In recent years, Re and Tc radiopharmaceutical chemistry with the tricarbonyl precursor *fac*-[*M*(CO)_3_(H_2_O)_3_]^+^ (*M* = ^99m^Tc, Re) has expanded continuously with the development of suitably derivatized novel ligand systems which efficiently displace the coordinating water mol­ecules to produce complexes with high *in vivo* stability, favorable pharmaco­kinetic properties, and target tissue specificity (Mundwiler *et al.*, 2004 ▸; Tri­antis *et al.*, 2013 ▸; Jürgens *et al.*, 2014 ▸; Alberto, 2012 ▸). In this article, we describe the crystal structure of a ‘2 + 1’ tricarbonyl rhenium(I) complex, *fac-*[*M*(CO)_3_(*L*)(NO-QA)], where *L* is tri­phenyl­arsane and NO-QA deprotonated quinaldic acid. This study is part of our ongoing research in the field of rhenium coordination compounds, particularly complexes bearing the *fac*-[Re(CO)_3_]^+^ synthon, to develop new mol­ecular radiopharmaceuticals. Related rhenium(I) tricarbonyl complexes have been reported by Schutte *et al.* (2011 ▸) and Manicum *et al.* (2015 ▸).
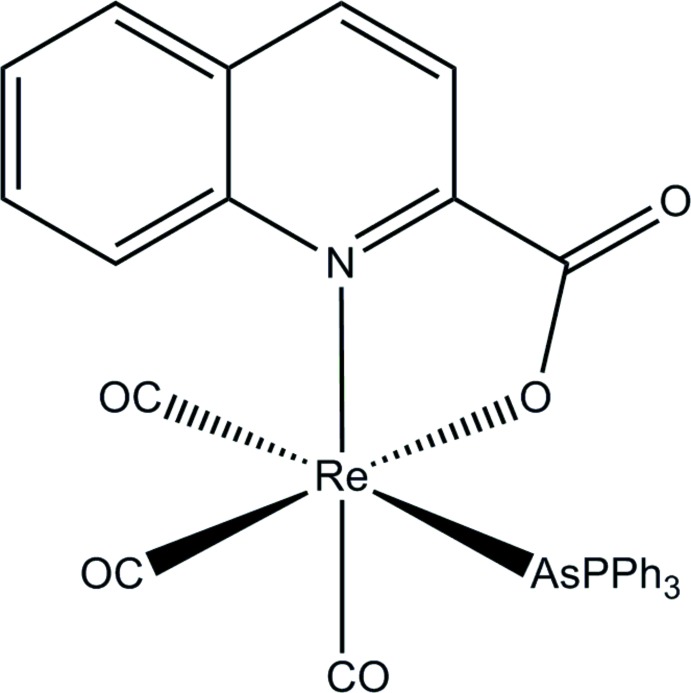


## Structural commentary   

**Figure 1:**
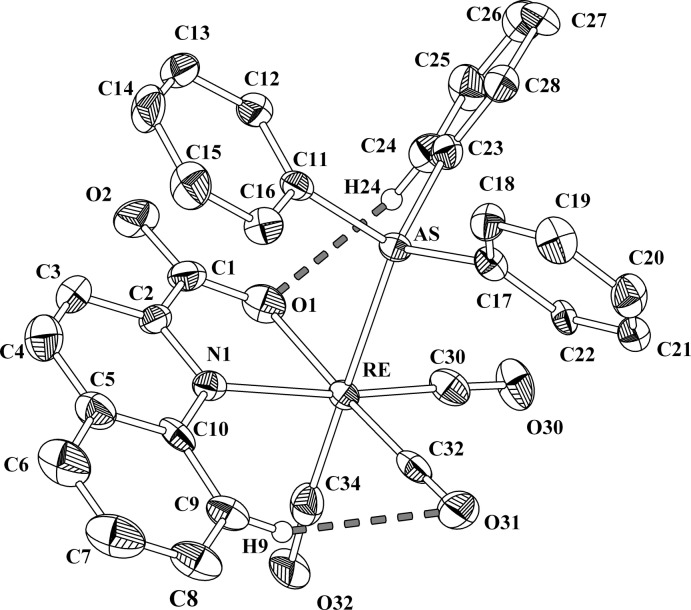
The mol­ecular structure and atom-labelling scheme of the title compound, with displacement ellipsoids drawn at the 50% probability level. H atoms have been omitted for clarity, except for those involved in intra­molecular hydrogen bonding (dashed grey lines).

In the title compound, the Re^I^ cation is in a distorted octa­hedral environment (Fig. 1 ▸). The apical positions of the octa­hedron are occupied by the monodentate arsane ligand and one of the carbonyl groups (C34 O32). The rhenium atom lies almost on the equatorial plane [displacement = 0.0459 (6) Å]. The five-membered ring defined by the metal ion and the chelating bidentate NO-QA anion is almost planar [maximum deviation of 0.078 (6) Å for atom N1]. One phenyl ring (C11–C16) of the tri­phenyl­arsane ligand exhibits intra­molecular π–π inter­action with the NO-QA ligand (Fig. 1 ▸), the distance from the centroid of the phenyl ring to the plane of the NO-QA ligand being 3.495 Å and the angle between the planes being 9.1°. In addition, intra­molecular hydrogen bonds are established between the phenyl rings of the NO-QA ligand (C9—H9⋯O31) and between one of the phenyl rings of the tri­phenyl­arsane ligand (C24—H24⋯O1) with one carbonyl oxygen atom and one carboxyl­ate oxygen atom respectively (Fig.1; Table 1 ▸). The Re—C O bond length in the apical position [Re—C34: 1.937 (12) Å] is longer than those in the equatorial plane [Re—C32 = 1.893 (8) Å and Re—C30 = 1.904 (9) Å] because of the *trans* influence of the tri­phenyl­arsane ligands, as expected (Coe & Glenwright, 2000 ▸; Otto & Johansson, 2002 ▸). Taking into account that this is the first structurally characterized Re^I^ tri­phenyl­arsane tricarbonyl complex, there are no other Re^I^ compound to compare with, but the measured Re—As distance of 2.5855 (10) Å is close to those given by Commons & Hoskins (1975 ▸) of 2.569–2.584 Å where the di(di­phenyl­arsino)methane ligand is coordinating to an Re^I^ ion.

## Supra­molecular features   

**Figure 2:**
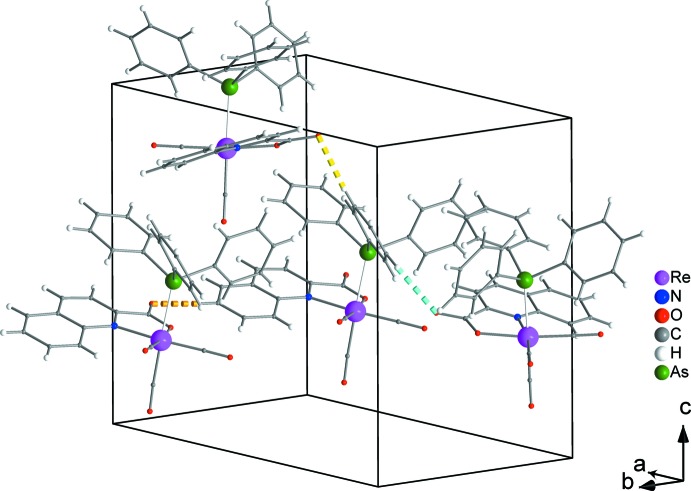
Weak inter­molecular hydrogen bonds (C7—H7⋯O2, C19—H19⋯O2 and C21—H21⋯O2) between neighbouring complexes indicated by dashed orange, yellow and turquoise lines, respectively. Intra­molecular hydrogen bonds are not shown for clarity.

**Figure 3:**
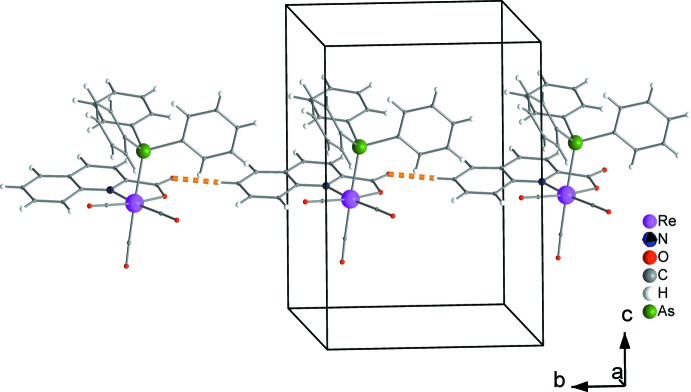
Chains of complexes, formed through C7—H7⋯O2 hydrogen bonds (dashed orange lines), parallel to the *b* axis.

**Figure 4:**
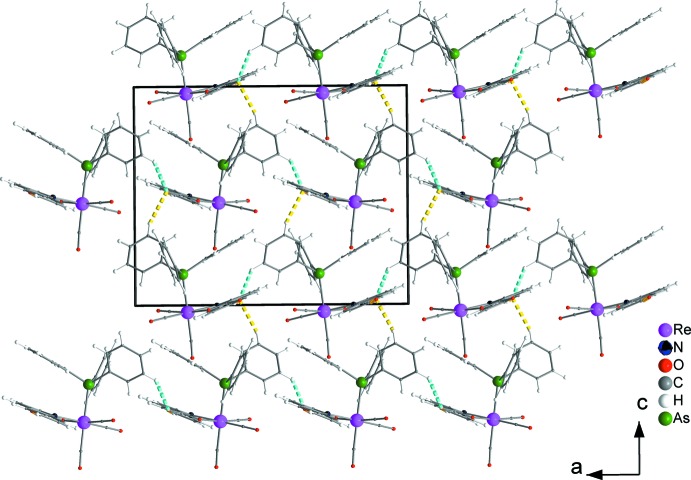
The three-dimensional network of neighbouring chains formed through C19—H19⋯O2 and C21—H21⋯O2 hydrogen bonds (dashed orange and dashed turquoise lines, respectively) in a view along the *b*-axis direction.

Weak inter­molecular hydrogen bonds (C7—H7⋯O2, C19—H19⋯O2 and C21—H21⋯O2, Table 1 ▸ and Fig. 2 ▸) are developed among the complexes in the crystal structure. Those of the C7—H7⋯O2 type result in chain formation parallel to the *b* axis (Fig. 3 ▸). Neighbouring chains further inter­act through C19—H19⋯O2 and C21—H21⋯O2 inter­actions and build up the three-dimensional set-up of the structure (Fig. 4 ▸).

## Synthesis and crystallization   

To a stirred solution of quinaldic acid (17.3 mg, 0.1 mmol) in 5 ml methanol, a solution of [NEt_4_]_2_[ReBr_3_(CO)_3_] (77 mg, 0.1 mmol) in 5 ml methanol was added. The mixture was heated at 323 K and after 30 min a solution of tri­phenyl­arsane (0.1 mmol) in 3 ml methanol was added. The mixture was stirred under reflux for 2 h and the reaction progress was monitored by HPLC. The solvent was removed under reduced pressure and the solid residue was recrystallized from a di­chloro­methane/methanol mixture. The resulting solid was redissolved in a minimum volume of di­chloro­methane, layered with methanol and left to stand at room temperature. After several days crystals suitable for X-ray analysis were isolated (yield: 46.8 mg, 60%).

## Refinement   

Crystal data, data collection and structure refinement details are summarized in Table 2 ▸. C-bound H atoms were placed in idealized positions and refined using a riding model with C—H = 0.95 Å and *U*_iso_(H) = 1.2*U*_eq_(C).

## Supplementary Material

Crystal structure: contains datablock(s) I, New_Global_Publ_Block. DOI: 10.1107/S2056989015024640/wm5246sup1.cif

Structure factors: contains datablock(s) I. DOI: 10.1107/S2056989015024640/wm5246Isup2.hkl

CCDC reference: 1443806

Additional supporting information:  crystallographic information; 3D view; checkCIF report

## Figures and Tables

**Table 1:** Hydrogen-bond geometry (Å, °)

*D*—H⋯*A*	*D*—H	H⋯*A*	*D*⋯*A*	*D*—H⋯*A*
C9—H9⋯O31	0.95	2.60	3.431 (11)	146
C24—H24⋯O1	0.95	2.47	3.276 (12)	143
C7—H7⋯O2^i^	0.95	2.20	3.151 (11)	176
C21—H21⋯O2^ii^	0.95	2.57	3.251 (11)	128
C19—H19⋯O2^iii^	0.95	2.46	3.337 (11)	153

Symmetry codes: (i) 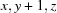; (ii) 
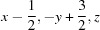
; (iii) 
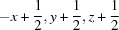
.

**Table 2:** Experimental details

Crystal data	
Chemical formula	[Re(C_10_H_6_NO_2_)(C_18_H_15_As)(CO)_3_]
*M*_r_	748.61
Crystal system, space group	Orthorhombic, *P* *n* *a*2_1_
Temperature (K)	160
*a*, *b*, *c* (Å)	18.1637 (3), 10.3463 (2), 14.5322 (3)
*V* (Å^3^)	2730.99 (9)
*Z*	4
Radiation type	Cu *K*α
μ (mm^−1^)	10.40
Crystal size (mm)	0.27 × 0.27 × 0.09

Data collection	
Diffractometer	Rigaku R-AXIS SPIDER IPDS diffractometer
Absorption correction	Multi-scan (*CrystalClear*; Rigaku, 2005 ▸)
*T*_min_, *T*_max_	0.443, 1.00
No. of measured, independent and observed [*I* > 2σ(*I*)] reflections	16386, 4768, 4655
*R*_int_	0.052
(sin θ/λ)_max_ (Å^−1^)	0.599

Refinement	
*R*[*F* ^2^ > 2σ(*F* ^2^)], *wR*(*F* ^2^), *S*	0.033, 0.076, 1.05
No. of reflections	4768
No. of parameters	352
No. of restraints	1
H-atom treatment	H-atom parameters constrained
Δρ_max_, Δρ_min_ (e Å^−3^)	1.39, −1.50
Absolute structure	Flack *x* determined using 2096 quotients [(*I* ^+^)−(*I* ^−^)]/[(*I* ^+^)+(*I* ^−^)] (Parsons *et al.*, 2013 ▸)
Absolute structure parameter	0.019 (7)

Computer programs: *CrystalClear* (Rigaku, 2005 ▸), *SHELXS97* (Sheldrick, 2008 ▸), *SHELXL2014* (Sheldrick, 2015 ▸), *DIAMOND* (Crystal Impact, 2012 ▸) and *PLATON* (Spek, 2009 ▸).

## supplementary crystallographic information

### Crystal data

**Table:**

[Re(C_10_H_6_NO_2_)(C_18_H_15_As)(CO)_3_]	*D*_x_ = 1.821 Mg m^−^^3^
*M**_r_* = 748.61	Cu *K*α radiation, λ = 1.54178 Å
Orthorhombic, *P**n**a*2_1_	Cell parameters from 17073 reflections
*a* = 18.1637 (3) Å	θ = 6.6–71.9°
*b* = 10.3463 (2) Å	µ = 10.40 mm^−^^1^
*c* = 14.5322 (3) Å	*T* = 160 K
*V* = 2730.99 (9) Å^3^	Parallelepided, colorless
*Z* = 4	0.27 × 0.27 × 0.09 mm
*F*(000) = 1448	

### Data collection

**Table:**

Rigaku R-AXIS SPIDER IPDS diffractometer	4655 reflections with *I* > 2σ(*I*)
Radiation source: fine-focus sealed tube	*R*_int_ = 0.052
θ scans	θ_max_ = 67.5°, θ_min_ = 7.2°
Absorption correction: multi-scan (*CrystalClear*; Rigaku, 2005)	*h* = −20→15
*T*_min_ = 0.443, *T*_max_ = 1.00	*k* = −12→8
16386 measured reflections	*l* = −17→17
4768 independent reflections	

### Refinement

**Table:**

Refinement on *F*^2^	Hydrogen site location: inferred from neighbouring sites
Least-squares matrix: full	H-atom parameters constrained
*R*[*F*^2^ > 2σ(*F*^2^)] = 0.033	*w* = 1/[σ^2^(*F*_o_^2^) + (0.0343*P*)^2^ + 0.6895*P*] where *P* = (*F*_o_^2^ + 2*F*_c_^2^)/3
*wR*(*F*^2^) = 0.076	(Δ/σ)_max_ = 0.001
*S* = 1.05	Δρ_max_ = 1.39 e Å^−^^3^
4768 reflections	Δρ_min_ = −1.50 e Å^−^^3^
352 parameters	Absolute structure: Flack *x* determined using 2096 quotients [(*I*^+^)-(*I*^-^)]/[(*I*^+^)+(*I*^-^)] (Parsons *et al.*, 2013)
1 restraint	Absolute structure parameter: 0.019 (7)

### Special details

**Table:**

Geometry. All e.s.d.'s (except the e.s.d. in the dihedral angle between two l.s. planes) are estimated using the full covariance matrix. The cell e.s.d.'s are taken into account individually in the estimation of e.s.d.'s in distances, angles and torsion angles; correlations between e.s.d.'s in cell parameters are only used when they are defined by crystal symmetry. An approximate (isotropic) treatment of cell e.s.d.'s is used for estimating e.s.d.'s involving l.s. planes.

### Fractional atomic coordinates and isotropic or equivalent isotropic displacement parameters (Å^2^)

**Table:**

	*x*	*y*	*z*	*U*_iso_*/*U*_eq_	
Re	0.19141 (2)	0.85298 (3)	0.46794 (4)	0.01930 (12)	
N1	0.2993 (4)	0.9464 (7)	0.4983 (4)	0.0229 (17)	
O1	0.2681 (3)	0.6990 (5)	0.4781 (5)	0.0335 (14)	
O2	0.3818 (4)	0.6409 (5)	0.5218 (5)	0.0385 (18)	
C1	0.3333 (5)	0.7230 (9)	0.5073 (6)	0.029 (2)	
C2	0.3522 (5)	0.8626 (7)	0.5221 (6)	0.0226 (19)	
C3	0.4205 (5)	0.9006 (10)	0.5543 (7)	0.038 (2)	
H3	0.4555	0.8369	0.5719	0.046*	
C4	0.4377 (6)	1.0254 (10)	0.5610 (7)	0.040 (2)	
H4	0.4844	1.0513	0.5840	0.048*	
C5	0.3859 (6)	1.1180 (9)	0.5337 (7)	0.035 (2)	
C6	0.4030 (7)	1.2520 (9)	0.5344 (7)	0.049 (3)	
H6	0.4499	1.2802	0.5554	0.059*	
C7	0.3516 (8)	1.3419 (9)	0.5048 (8)	0.054 (3)	
H7	0.3624	1.4317	0.5079	0.064*	
C8	0.2841 (6)	1.2999 (7)	0.4704 (9)	0.040 (2)	
H8	0.2497	1.3621	0.4489	0.048*	
C9	0.2659 (5)	1.1707 (7)	0.4666 (8)	0.034 (2)	
H9	0.2200	1.1438	0.4419	0.040*	
C10	0.3176 (5)	1.0772 (9)	0.5008 (6)	0.027 (2)	
As	0.17369 (5)	0.81237 (9)	0.64209 (6)	0.0203 (2)	
C11	0.2613 (5)	0.8487 (7)	0.7149 (6)	0.024 (2)	
C12	0.3060 (4)	0.7508 (10)	0.7455 (6)	0.030 (2)	
H12	0.2925	0.6633	0.7349	0.037*	
C13	0.3712 (5)	0.7793 (10)	0.7920 (6)	0.039 (2)	
H13	0.4019	0.7115	0.8137	0.047*	
C14	0.3907 (6)	0.9064 (10)	0.8063 (7)	0.042 (3)	
H14	0.4353	0.9260	0.8375	0.050*	
C15	0.3456 (6)	1.0064 (11)	0.7755 (7)	0.042 (3)	
H15	0.3586	1.0938	0.7873	0.050*	
C16	0.2817 (5)	0.9779 (9)	0.7274 (6)	0.029 (2)	
H16	0.2521	1.0454	0.7031	0.035*	
C17	0.0961 (5)	0.9123 (8)	0.6992 (6)	0.0245 (19)	
C18	0.1054 (5)	0.9746 (8)	0.7841 (5)	0.0279 (19)	
H18	0.1501	0.9653	0.8173	0.033*	
C19	0.0488 (5)	1.0500 (9)	0.8192 (6)	0.036 (2)	
H19	0.0552	1.0959	0.8753	0.043*	
C20	−0.0172 (5)	1.0575 (9)	0.7717 (7)	0.035 (2)	
H20	−0.0562	1.1083	0.7960	0.042*	
C21	−0.0273 (5)	0.9923 (8)	0.6896 (6)	0.029 (2)	
H21	−0.0731	0.9970	0.6581	0.035*	
C22	0.0299 (4)	0.9204 (8)	0.6543 (6)	0.0208 (17)	
H22	0.0233	0.8757	0.5978	0.025*	
C23	0.1471 (5)	0.6365 (7)	0.6769 (6)	0.027 (2)	
C24	0.1601 (5)	0.5348 (8)	0.6156 (7)	0.033 (2)	
H24	0.1829	0.5514	0.5580	0.040*	
C25	0.1398 (5)	0.4106 (9)	0.6385 (8)	0.038 (2)	
H25	0.1484	0.3411	0.5972	0.046*	
C26	0.1067 (6)	0.3891 (9)	0.7223 (8)	0.043 (3)	
H26	0.0927	0.3036	0.7384	0.052*	
C27	0.0935 (5)	0.4873 (9)	0.7831 (7)	0.042 (2)	
H27	0.0696	0.4697	0.8399	0.050*	
C28	0.1148 (5)	0.6126 (9)	0.7618 (6)	0.032 (2)	
H28	0.1075	0.6808	0.8046	0.039*	
C30	0.1086 (5)	0.7452 (9)	0.4440 (5)	0.031 (2)	
O30	0.0571 (4)	0.6812 (7)	0.4282 (5)	0.0404 (17)	
C32	0.1253 (4)	0.9943 (7)	0.4725 (7)	0.0262 (17)	
O31	0.0843 (3)	1.0805 (6)	0.4766 (6)	0.0421 (16)	
C34	0.2037 (5)	0.8780 (10)	0.3367 (8)	0.033 (2)	
O32	0.2073 (4)	0.8903 (9)	0.2592 (5)	0.047 (2)	

### Atomic displacement parameters (Å^2^)

**Table:**

	*U*^11^	*U*^22^	*U*^33^	*U*^12^	*U*^13^	*U*^23^
Re	0.0220 (2)	0.01733 (18)	0.01854 (19)	−0.00165 (12)	0.00153 (18)	−0.00057 (19)
N1	0.026 (4)	0.022 (4)	0.020 (4)	0.003 (3)	0.002 (3)	0.001 (3)
O1	0.044 (4)	0.022 (3)	0.035 (3)	−0.004 (3)	0.000 (4)	−0.002 (3)
O2	0.044 (5)	0.026 (3)	0.046 (4)	0.018 (3)	0.004 (3)	0.004 (3)
C1	0.033 (6)	0.028 (5)	0.026 (4)	0.007 (4)	0.000 (4)	0.005 (4)
C2	0.022 (5)	0.025 (4)	0.020 (4)	0.004 (4)	−0.002 (4)	0.002 (3)
C3	0.026 (6)	0.045 (6)	0.044 (6)	0.003 (5)	−0.004 (5)	0.011 (5)
C4	0.033 (6)	0.041 (6)	0.046 (6)	−0.010 (5)	−0.005 (4)	−0.004 (5)
C5	0.040 (6)	0.030 (5)	0.035 (5)	−0.008 (5)	0.001 (4)	0.005 (4)
C6	0.066 (8)	0.033 (5)	0.050 (6)	−0.028 (6)	−0.007 (6)	−0.002 (5)
C7	0.084 (11)	0.029 (5)	0.048 (7)	−0.018 (6)	−0.004 (7)	0.003 (5)
C8	0.065 (7)	0.018 (4)	0.037 (5)	−0.001 (4)	0.011 (7)	0.001 (6)
C9	0.045 (6)	0.024 (4)	0.033 (4)	−0.005 (4)	0.005 (6)	0.006 (6)
C10	0.034 (6)	0.023 (4)	0.024 (4)	−0.013 (4)	0.007 (3)	0.003 (4)
As	0.0243 (5)	0.0170 (4)	0.0196 (4)	−0.0012 (4)	0.0007 (4)	0.0009 (4)
C11	0.027 (5)	0.024 (4)	0.021 (4)	0.001 (3)	0.004 (4)	0.004 (3)
C12	0.030 (6)	0.029 (5)	0.032 (5)	−0.003 (4)	−0.001 (4)	0.006 (4)
C13	0.032 (5)	0.054 (6)	0.032 (5)	0.007 (5)	−0.003 (4)	0.011 (5)
C14	0.024 (6)	0.056 (6)	0.045 (6)	−0.011 (5)	−0.010 (5)	−0.004 (6)
C15	0.038 (6)	0.050 (7)	0.038 (6)	−0.016 (6)	−0.008 (5)	−0.009 (5)
C16	0.033 (6)	0.026 (4)	0.028 (5)	−0.001 (4)	−0.003 (4)	−0.004 (4)
C17	0.027 (5)	0.021 (4)	0.026 (4)	−0.002 (4)	0.006 (4)	0.000 (4)
C18	0.026 (5)	0.039 (5)	0.019 (4)	−0.001 (4)	−0.002 (4)	−0.006 (4)
C19	0.041 (6)	0.036 (5)	0.030 (5)	−0.004 (4)	0.006 (4)	−0.015 (4)
C20	0.030 (6)	0.032 (5)	0.043 (6)	0.007 (5)	0.009 (4)	−0.004 (4)
C21	0.025 (5)	0.035 (5)	0.028 (5)	−0.004 (4)	0.000 (4)	0.005 (4)
C22	0.018 (4)	0.027 (4)	0.018 (4)	−0.008 (3)	0.003 (4)	−0.003 (4)
C23	0.022 (5)	0.026 (5)	0.031 (5)	−0.004 (4)	−0.002 (4)	0.002 (3)
C24	0.038 (6)	0.022 (5)	0.039 (6)	0.001 (4)	−0.002 (4)	−0.002 (4)
C25	0.035 (6)	0.023 (4)	0.057 (7)	−0.010 (4)	0.001 (6)	−0.002 (5)
C26	0.036 (6)	0.023 (4)	0.071 (8)	−0.011 (5)	−0.010 (6)	0.014 (5)
C27	0.037 (6)	0.047 (6)	0.041 (6)	−0.007 (5)	0.004 (5)	0.024 (5)
C28	0.028 (5)	0.033 (5)	0.037 (5)	0.001 (4)	−0.001 (4)	0.004 (4)
C30	0.040 (6)	0.032 (4)	0.021 (5)	−0.004 (4)	0.006 (4)	−0.002 (3)
O30	0.037 (4)	0.045 (4)	0.039 (4)	−0.022 (3)	0.003 (3)	−0.014 (3)
C32	0.032 (4)	0.027 (4)	0.019 (3)	−0.014 (4)	0.003 (4)	0.003 (5)
O31	0.039 (4)	0.038 (3)	0.049 (4)	0.017 (3)	0.009 (4)	0.012 (4)
C34	0.025 (5)	0.030 (5)	0.044 (7)	−0.005 (4)	−0.002 (5)	−0.002 (5)
O32	0.041 (4)	0.086 (6)	0.015 (4)	−0.004 (4)	0.008 (3)	0.009 (4)

### Geometric parameters (Å, º)

**Table:**

Re—C32	1.893 (8)	C13—C14	1.378 (14)
Re—C30	1.904 (9)	C13—H13	0.9500
Re—C34	1.937 (12)	C14—C15	1.394 (14)
Re—O1	2.122 (6)	C14—H14	0.9500
Re—N1	2.229 (7)	C15—C16	1.387 (13)
Re—As	2.5855 (10)	C15—H15	0.9500
N1—C2	1.339 (10)	C16—H16	0.9500
N1—C10	1.394 (11)	C17—C22	1.371 (11)
O1—C1	1.281 (11)	C17—C18	1.403 (11)
O2—C1	1.243 (10)	C18—C19	1.388 (12)
C1—C2	1.500 (11)	C18—H18	0.9500
C2—C3	1.383 (12)	C19—C20	1.386 (13)
C3—C4	1.333 (12)	C19—H19	0.9500
C3—H3	0.9500	C20—C21	1.383 (12)
C4—C5	1.400 (13)	C20—H20	0.9500
C4—H4	0.9500	C21—C22	1.377 (11)
C5—C10	1.395 (13)	C21—H21	0.9500
C5—C6	1.422 (12)	C22—H22	0.9500
C6—C7	1.386 (17)	C23—C28	1.388 (12)
C6—H6	0.9500	C23—C24	1.399 (11)
C7—C8	1.394 (17)	C24—C25	1.377 (11)
C7—H7	0.9500	C24—H24	0.9500
C8—C9	1.378 (10)	C25—C26	1.376 (15)
C8—H8	0.9500	C25—H25	0.9500
C9—C10	1.435 (13)	C26—C27	1.367 (14)
C9—H9	0.9500	C26—H26	0.9500
As—C17	1.935 (9)	C27—C28	1.389 (12)
As—C11	1.947 (10)	C27—H27	0.9500
As—C23	1.949 (8)	C28—H28	0.9500
C11—C12	1.373 (12)	C30—O30	1.169 (10)
C11—C16	1.399 (11)	C32—O31	1.163 (9)
C12—C13	1.395 (11)	C34—O32	1.136 (12)
C12—H12	0.9500		
			
C32—Re—C30	87.6 (3)	C12—C11—As	121.1 (6)
C32—Re—C34	90.3 (4)	C16—C11—As	118.2 (6)
C30—Re—C34	89.4 (4)	C11—C12—C13	120.2 (9)
C32—Re—O1	173.8 (4)	C11—C12—H12	119.9
C30—Re—O1	95.2 (3)	C13—C12—H12	119.9
C34—Re—O1	95.3 (3)	C14—C13—C12	119.6 (9)
C32—Re—N1	102.5 (3)	C14—C13—H13	120.2
C30—Re—N1	169.8 (3)	C12—C13—H13	120.2
C34—Re—N1	92.0 (3)	C13—C14—C15	120.6 (9)
O1—Re—N1	74.6 (2)	C13—C14—H14	119.7
C32—Re—As	90.7 (3)	C15—C14—H14	119.7
C30—Re—As	89.1 (2)	C16—C15—C14	119.7 (9)
C34—Re—As	178.2 (3)	C16—C15—H15	120.1
O1—Re—As	83.80 (19)	C14—C15—H15	120.1
N1—Re—As	89.23 (17)	C15—C16—C11	119.4 (9)
C2—N1—C10	116.8 (7)	C15—C16—H16	120.3
C2—N1—Re	113.6 (6)	C11—C16—H16	120.3
C10—N1—Re	129.4 (6)	C22—C17—C18	119.7 (8)
C1—O1—Re	118.9 (5)	C22—C17—As	117.9 (6)
O2—C1—O1	125.4 (9)	C18—C17—As	122.4 (7)
O2—C1—C2	118.1 (9)	C19—C18—C17	119.5 (8)
O1—C1—C2	116.5 (8)	C19—C18—H18	120.2
N1—C2—C3	123.2 (8)	C17—C18—H18	120.2
N1—C2—C1	115.0 (8)	C20—C19—C18	119.3 (8)
C3—C2—C1	121.8 (8)	C20—C19—H19	120.4
C4—C3—C2	120.7 (9)	C18—C19—H19	120.4
C4—C3—H3	119.7	C21—C20—C19	121.2 (9)
C2—C3—H3	119.7	C21—C20—H20	119.4
C3—C4—C5	119.0 (9)	C19—C20—H20	119.4
C3—C4—H4	120.5	C22—C21—C20	119.0 (9)
C5—C4—H4	120.5	C22—C21—H21	120.5
C10—C5—C4	119.3 (9)	C20—C21—H21	120.5
C10—C5—C6	119.5 (10)	C17—C22—C21	121.2 (8)
C4—C5—C6	121.2 (10)	C17—C22—H22	119.4
C7—C6—C5	120.3 (11)	C21—C22—H22	119.4
C7—C6—H6	119.9	C28—C23—C24	120.2 (8)
C5—C6—H6	119.9	C28—C23—As	120.1 (6)
C6—C7—C8	119.7 (9)	C24—C23—As	119.7 (7)
C6—C7—H7	120.2	C25—C24—C23	120.2 (9)
C8—C7—H7	120.2	C25—C24—H24	119.9
C9—C8—C7	121.8 (10)	C23—C24—H24	119.9
C9—C8—H8	119.1	C26—C25—C24	118.8 (10)
C7—C8—H8	119.1	C26—C25—H25	120.6
C8—C9—C10	118.9 (9)	C24—C25—H25	120.6
C8—C9—H9	120.6	C27—C26—C25	121.9 (9)
C10—C9—H9	120.6	C27—C26—H26	119.1
N1—C10—C5	120.9 (9)	C25—C26—H26	119.1
N1—C10—C9	119.3 (8)	C26—C27—C28	120.1 (9)
C5—C10—C9	119.8 (8)	C26—C27—H27	120.0
C17—As—C11	105.0 (4)	C28—C27—H27	120.0
C17—As—C23	102.0 (4)	C23—C28—C27	118.8 (9)
C11—As—C23	104.0 (4)	C23—C28—H28	120.6
C17—As—Re	115.1 (3)	C27—C28—H28	120.6
C11—As—Re	113.5 (3)	O30—C30—Re	178.5 (8)
C23—As—Re	115.9 (3)	O31—C32—Re	179.0 (9)
C12—C11—C16	120.4 (9)	O32—C34—Re	176.5 (10)

### Hydrogen-bond geometry (Å, º)

**Table:**

*D*—H···*A*	*D*—H	H···*A*	*D*···*A*	*D*—H···*A*
C9—H9···O31	0.95	2.60	3.431 (11)	146
C24—H24···O1	0.95	2.47	3.276 (12)	143
C7—H7···O2^i^	0.95	2.20	3.151 (11)	176
C21—H21···O2^ii^	0.95	2.57	3.251 (11)	128
C19—H19···O2^iii^	0.95	2.46	3.337 (11)	153

Symmetry codes: (i) *x*, *y*+1, *z*; (ii) *x*−1/2, −*y*+3/2, *z*; (iii) −*x*+1/2, *y*+1/2, *z*+1/2.

## References

[bb1] Alberto, R. (2012). *Cosmos*, **8**, 83–101.

[bb2] Coe, J. B. & Glenwright, J. S. (2000). *Coord. Chem. Rev.* **203**, 5–80.

[bb3] Commons, C. J. & Hoskins, B. F. (1975). *Aust. J. Chem.* **28**, 1201.

[bb4] Crystal Impact (2012). *DIAMOND.* Crystal Impact GbR, Bonn, Germany.

[bb5] Jürgens, S., Herrmann, W. & Kühn, F. (2014). *J. Organomet. Chem.* **751**, 83–89.

[bb6] Manicum, A.-L., Visser, H., Engelbrecht, I. & Roodt, A. (2015). *Z. Kristallogr.* **230**, 150–152.

[bb7] Mundwiler, S., Kündig, M., Ortner, K. & Alberto, R. (2004). *Dalton Trans.* pp. 1320. 10.1039/b400220b 15252624

[bb8] Otto, S. & Johansson, H. M. (2002). *Inorg. Chim. Acta*, **329**, 135–140.

[bb9] Parsons, S., Flack, H. D. & Wagner, T. (2013). *Acta Cryst.* B**69**, 249–259. 10.1107/S2052519213010014 PMC3661305 23719469

[bb10] Rigaku (2005). *CrystalClear*. Rigaku MSC, The Woodlands, Texas, USA.

[bb11] Schutte, M., Kemp, G., Visser, H. & Roodt, A. (2011). *Inorg. Chem.* **50**, 12486–12498. 10.1021/ic2013792 22111710

[bb12] Sheldrick, G. M. (2008). *Acta Cryst.* A**64**, 112–122. 10.1107/S0108767307043930 18156677

[bb13] Sheldrick, G. M. (2015). *Acta Cryst.* C**71**, 3–8.

[bb14] Spek, A. L. (2009). *Acta Cryst.* D**65**, 148–155. 10.1107/S090744490804362X PMC2631630 19171970

[bb15] Triantis, C., Tsotakos, T., Tsoukalas, C., Sagnou, M., Raptopoulou, C. P., Terzis, A., Psycharis, V., Pelecanou, M., Pirmettis, I. & Papadopoulos, M. (2013). *Inorg. Chem.* **52**, 12995–13003. 10.1021/ic401503b 24199833

